# Fatal *Strongyloides stercoralis* hyperinfection syndrome in an alcoholic diabetic patient from México

**DOI:** 10.7705/biomedica.5071

**Published:** 2020-08-20

**Authors:** Elba G. Rodríguez-Pérez, Alma Y. Arce-Mendoza, Roberto Saldívar-Palacios, Kevin Escandón-Vargas

**Affiliations:** 1 Parasitología Clínica, Hospital Universitario Dr. José Eleuterio González, Universidad Autónoma de Nuevo León, Monterrey, México Universidad Autónoma de Nuevo León Universidad Autónoma de Nuevo León Monterrey Mexico; 2 Departamento de Inmunología, Facultad de Medicina, Universidad Autónoma de Nuevo León, Monterrey, México Universidad Autónoma de Nuevo León Departamento de Inmunología Facultad de Medicina Universidad Autónoma de Nuevo León Monterrey Mexico; 3 Departamento de Microbiología, Facultad de Medicina, Universidad Autónoma de Nuevo León, Monterrey, México Universidad Autónoma de Nuevo León Departamento de Microbiología Facultad de Medicina Universidad Autónoma de Nuevo León Monterrey Mexico; 4 Escuela de Medicina, Universidad del Valle, Cali, Colombia Universidad del Valle Universidad del Valle Cali Colombia

**Keywords:** Strongyloides stercoralis, strongyloidiasis, neglected diseases, México, Strongyloides stercoralis, estrongiloidiasis, enfermedades desatendidas, México

## Abstract

*Strongyloides stercoralis* hyperinfection syndrome is a medical emergency that requires a high level of suspicion. Immunocompromised patients are at high risk of hyperinfection syndrome; however, malnutrition, alcoholism, and diabetes mellitus also need to be considered as predisposing factors. The diagnosis and treatment of *Strongyloides* hyperinfection are challenging and patients often have severe complications. Consequently, mortality is overwhelmingly high, with proportions above 60%.

Herein, we report a case of *Strongyloides* hyperinfection in a 40-year-old alcoholic diabetic patient living in México. Unfortunately, the late diagnosis resulted in his death despite the treatment and supportive measures. Increased awareness is needed to prevent the dire consequences of strongyloidiasis.

## Resumen

El síndrome de hiperinfección por *Strongyloides stercoralis* es una emergencia médica que requiere una aguda sospecha clínica. Los pacientes inmunocomprometidos tienen alto riesgo de sufrir el síndrome de hiperinfección; sin embargo, la desnutrición, el alcoholismo y la diabetes mellitus también deben considerarse factores predisponentes. El diagnóstico y el tratamiento de la hiperinfección por *S. stercoralis* constituyen un desafío y los pacientes a menudo tienen complicaciones graves. Como consecuencia, la mortalidad es abrumadoramente alta, con proporciones superiores al 60 %.

Se presenta un caso de hiperinfección por *S. stercoralis* en un paciente diabético y alcohólico de 40 años que vivía en México. Infortunadamente, el diagnóstico tardío causó su muerte a pesar del tratamiento y las medidas de soporte. Se necesita un mayor conocimiento para prevenir las terribles consecuencias de la estrongiloidiasis.

Strongyloidiasis is a neglected parasitic disease mainly caused by *Strongyloides stercoralis* and, to a lesser extent, by *S. fuelleborni*. It is estimated to affect at least 370 million people worldwide, especially in tropical and subtropical regions ^1^^,^^2^. Although there is a lack of updated and comprehensive epidemiological data for *S. stercoralis*, strongyloidiasis is considered to be largely endemic to México. In a few community- and hospital-based surveys, the prevalence of *S. stercoralis* has been estimated from 0.1% to 68% ^3^^-^^5^.

*Strongyloides stercoralis* has a complex life cycle with both free-living and parasitic developmental stages including autoinfection ^1^^,^^6^^,^^7^. Rhabditiform larvae (L_1_, usual diagnostic stage) are passed in the stool after hatching from eggs deposited in the intestinal mucosa of an infected definitive host (human or dog). These larvae develop directly or indirectly into filariform larvae (L_3_, infective stage) that penetrate the intact skin after contact with contaminated soil. Filariform larvae migrate via the bloodstream and lymphatics to the lungs from where they ascend the tracheobronchial tree and are swallowed to reach the gastrointestinal tract. In the small intestine, larvae mature into adult female worms, which live embedded in the submucosa and produce eggs via parthenogenesis yielding rhabditiform larvae. These larvae can either be excreted in the stool or can become filariform larvae and penetrate intestinal mucosa or perianal skin resulting in autoinfection.

The unique life cycle of *S. stercoralis* is determinant for the presentation forms in infected persons. Infection ranges from asymptomatic to chronic symptomatic strongyloidiasis and severe or disseminated forms of the disease ^7^. Herein we report a fatal case of *S. stercoralis* hyperinfection in a hypoalbuminemic, alcoholic, and diabetic patient from México.

## Case presentation

A 40-year-old Mexican-born male with a history of homelessness was admitted to a hospital in Monterrey, México, for abdominal pain and vomiting of 2 weeks’ duration. The patient complained of a 10 kg weight loss in the previous month. He originally came from a low-income area in Oaxaca and had been living in Monterrey for the last 5 years. He had a history of alcoholism and diabetes mellitus. He did not take any medications.

On examination, he was afebrile, tachycardic, dehydrated, and had normal oxygen saturation. The pulmonary examination was unremarkable. He had a distended, slightly tender abdomen with diminished bowel sounds. His white blood cell count was 16,200/µl with 92.3% neutrophils and 0.66% eosinophils (107/µl). Hemoglobin was 10.4 g/dl, and plasma glucose was 133 mg/dl.

Hyponatremia and hypokalemia were recorded. Serum total proteins and albumin were low (5.3 g/dl and 2.3 g/dl, respectively). An abdominal X-ray showed dilated small bowel loops suggestive of paralytic ileus. The initial treatment consisted of fluid resuscitation and placement of a nasogastric tube. On hospital day 3, his persistent ileus prompted the surgery service to perform a laparotomy.

**Figure 1:**
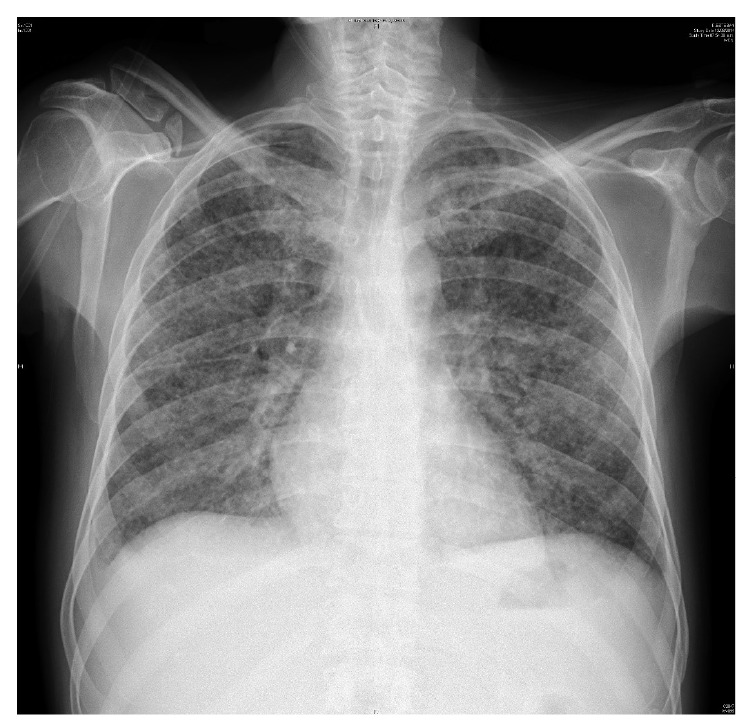
Chest x-ray showing bilateral diffuse interstitial infiltrates

Over the next 48 hours, the patient presented shortness of breath, hemoptysis, and fever. A chest X-ray revealed bilateral diffuse interstitial infiltrates (figure 1), and the patient was treated empirically with imipenem/ cilastatin and vancomycin for presumed bacteremia.

**Figure 2:**
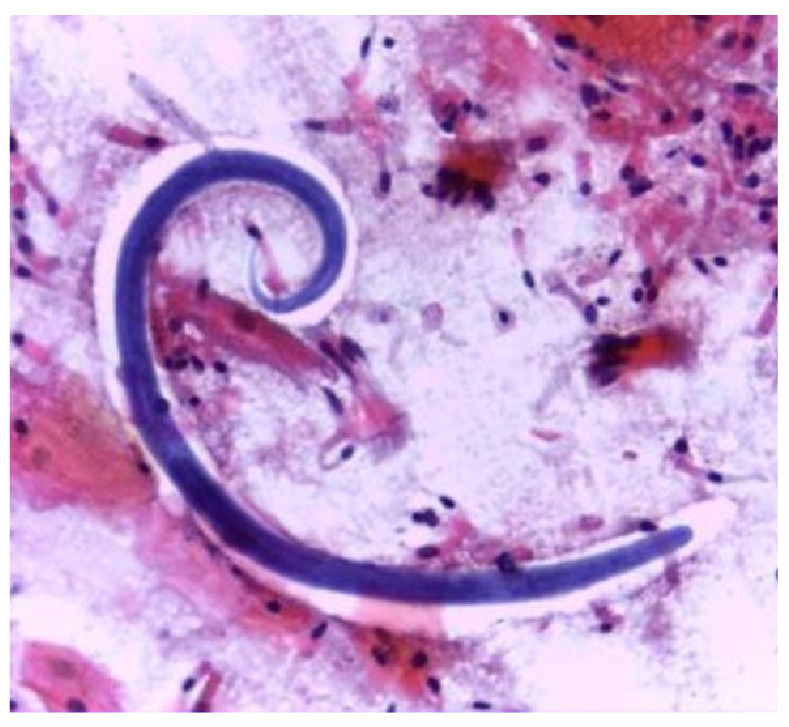
Bronchoalveolar lavage specimen showing a *Strongyloides stercoralis* filariform larva. Haematoxylin and eosin, 100X.

**Figure 3:**
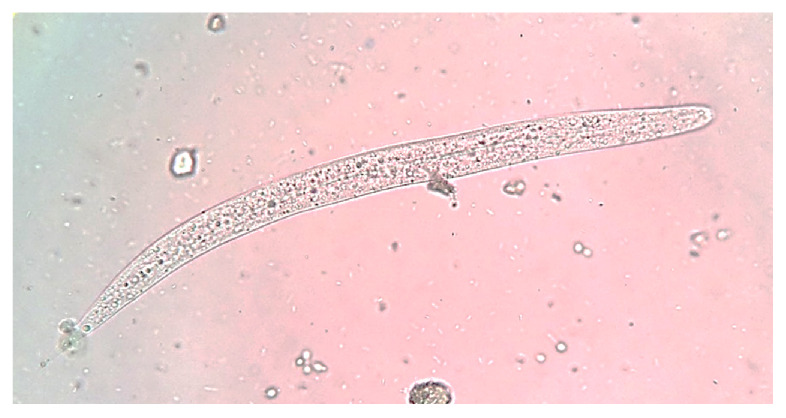
Wet mount of stool showing a *Strongyloides stercoralis* rhabditiform larva. Rhabditoid esophagus can be appreciated. Methylene blue, 100X.

Due to clinical worsening, he underwent bronchoscopy with bronchoalveolar lavage, which yielded a bloody lavage fluid from the right middle lobe. The pathological examination revealed numerous *S. stercoralis* filariform larvae (figure 2). Subsequently, the stool examination confirmed the presence of rhabditiform larvae (figure 3). Human T-cell lymphotropic virus type-1 (HTLV-1) and human immunodeficiency virus (HIV) serologies were negative. *Strongyloides stercoralis* larvae from bronchoalveolar lavage and feces provided diagnostic proof of the hyperinfection syndrome.

On hospital day 10, the patient was started on oral ivermectin 12 mg daily; however, two days later, ivermectin was administered per rectum owing to reduced oral intake. Later that day, the patient was taken to surgery for repair of omental evisceration. Postoperatively, the patient continued to deteriorate and developed respiratory failure requiring mechanical ventilation. He was transferred to the intensive care unit where he died despite the treatment and supportive measures administered.

The informed consent for this publication and the accompanying images was obtained from the patient’s nearest relative.

## Discussion

Strongyloidiasis can present in a wide variety of forms. In immunocompetent hosts, *S. stercoralis* mostly causes asymptomatic chronic infections that may persist for decades because of the ability of the parasite to sustain itself by low-level autoinfection. Gastrointestinal and dermatologic complaints, when present, are usually mild and include abdominal pain, diarrhea, constipation, vomiting, anorexia, urticaria, and larva currens ^1^^,^^7^. In contrast, in immunocompromised hosts, *S. stercoralis* can cause severe, life-threatening conditions resulting from accelerated autoinfection: The hyperinfection syndrome in which numerous larvae are confined to the autoinfective cycle (gastrointestinal tract, peritoneum, and lungs) and disseminated strongyloidiasis, which is characterized by larval migration to multiple organs beyond the range of the autoinfective cycle including the liver, heart, kidneys, brain, and skin.

Cell-mediated immunosuppression can trigger the massive dissemination of *S. stercoralis* larvae. Several immunosuppression-related risk factors for *Strongyloides* hyperinfection have been described including the use of corticosteroids and other immunosuppressants, HTLV-1 infection, organ transplantation, and hematologic malignancies ^1^^,^^7^. Other predisposing factors are malnutrition, alcoholism, and diabetes mellitus ^1^^,^^8^^,^^9^. These three conditions were present in the patient whose case we report. Interestingly, it has been postulated that the increased predisposition to *S. stercoralis* infection in alcoholics is related to reduced intestinal motility and increasing endogenous cortisol levels resulting in a deficient type 2 T helper cells (Th2) immune response, as well as in ecdysteroid-mimicking effects ^8^^,^^10^.

The increased larval burden in hyperinfection syndrome explains the development or exacerbation of gastrointestinal and respiratory symptoms. Cough, dyspnea, wheezing, and hemoptysis are common. Fever and neurologic manifestations may be present and should prompt a search for sepsis and meningitis due to translocation of enteric bacteria facilitated by worm migration ^7^. Patients with *S. stercoralis* hyperinfection often have severe complications such as paralytic ileus, bowel obstruction, bacteremia, meningitis, respiratory failure, and multiorgan dysfunction ^1^. Consequently, mortality is overwhelmingly high with proportions above 60% ^1^^,^^7^^,^^11^^,^^12^.

The diagnosis of hyperinfection syndrome requires a high level of suspicion. The detection of *S. stercoralis* filariform larvae in sputum or bronchoalveolar lavage fluid is the hallmark of hyperinfection syndrome ^7^. Contrary to asymptomatic and chronic forms of strongyloidiasis, microscopic examination of stool in patients with hyperinfection has increased sensitivity and often reveals numerous larvae ^7^^,^^11^. As seen in our case, eosinophilia is frequently absent in the hyperinfection syndrome (66-75%) ^11^^,^^12^.

The treatment of choice is ivermectin (200 µg/kg/day) based on its efficacy and favorable side-effect profile. Oral daily dosing is recommended for at least 2 weeks after parasite clearance from stools ^7^^,^^13^. Because many patients are critically ill or experience paralytic ileus, enteral absorption may be impaired.

Therefore, unorthodox administration routes (subcutaneous and rectal) have been attempted as adjunct or alternative to oral ivermectin. However, parenteral ivermectin is currently only available as a veterinary preparation and is not licensed for human use ^14^. There is a need for improved access to parenteral ivermectin and more data from pharmacological studies and clinical trials to guide the treatment of severe strongyloidiasis.

In conclusion, *Strongyloides* hyperinfection syndrome is a medical emergency that requires a high level of suspicion. Immunocompromised patients are particularly at high risk of hyperinfection syndrome; however, malnutrition, alcoholism, and diabetes mellitus also need to be considered as predisposing factors. Since both diagnosis and treatment are challenging, patients frequently have poor outcomes.

Our patient lived in an endemic area and had multiple risk factors for *Strongyloides* hyperinfection. Unfortunately, his ileus worsened shortly after the admission and the diagnosis was late, resulting in fatality despite the treatment and supportive measures administered. This case highlights the importance of awareness and suspicion of strongyloidiasis in individuals with impaired immunity or underlying risk conditions, especially if present or past exposure to endemic areas is reported, as well as early diagnosis and treatment of the hyperinfection syndrome.

## References

[1] Olsen A, van Lieshout L, Marti H, Polderman T, Polman K, Steinmann P (2009). Strongyloidiasis - the most neglected of the neglected tropical diseases?. Trans R Soc Trop Med Hyg.

[2] Bisoffi Z, Buonfrate D, Montresor A, Requena-Méndez A, Muñoz J, Krolewiecki AJ (2013). Strongyloides stercoralis: A plea for action. PLoS Negl Trop Dis.

[3] Guarner J, Matilde-Nava T, Villaseñor-Flores R, Sánchez-Mejorada G (1997). Frequency of intestinal parasites in adult cancer patients in México. Arch Med Res.

[4] Faulkner CT, Borrego-García B, Logan MH, New JC, Patton S (2003). Prevalence of endoparasitic infection in children and its relation with cholera prevention efforts in México. Rev Panam Salud Pública.

[5] Carrada-Bravo T (2008). Strongyloides stercoralis: ciclo vital, cuadros clínicos, epidemiología, patología y terapéutica. Revista Latinoamericana de Patología Clínica y Medicina de Laboratorio.

[6] Viney ME, Lok JB (2015). The biology of Strongyloides spp.

[7] Krolewiecki A, Nutman TB (2019). Strongyloidiasis. Infect Dis Clin North Am.

[8] Teixeira MCA, Pacheco FTF, Souza JN, Silva MLS, Inês EJ, Soares NM (2016). Strongyloides stercoralis infection in alcoholic patients. Biomed Res Int.

[9] McGuire E, Welch C, Melzer M (2019). Is Strongyloides seropositivity associated with diabetes mellitus? A retrospective case-control study in an East London NHS Trust. Trans R Soc Trop Med Hyg.

[10] Silva MLS, de J Inês E, Souza AB da S., Dias VM dos S, Guimarães CM, Menezes ER (2016). Association between Strongyloides stercoralis infection and cortisol secretion in alcoholic patients. Acta Trop.

[11] Buonfrate D, Requena-Méndez A, Angheben A, Muñoz J, Gobbi F, van Den Ende J (2013). Severe strongyloidiasis: A systematic review of case reports. BMC Infect Dis.

[12] Geri G, Rabbat A, Mayaux J, Zafrani L, Chalumeau-Lemoine L, Guidet B (2015). Strongyloides stercoralis hyperinfection syndrome: A case series and a review of the literature. Infection.

[13] Henríquez-Camacho C, Gotuzzo E, Echevarría J, White AC, Terashima A, Samalvides F (2016). Ivermectin versus albendazole or thiabendazole for Strongyloides stercoralis infection. Cochrane Database Syst Rev.

[14] Barrett J, Broderick C, Soulsby H, Wade P, Newsholme W (2016). Subcutaneous ivermectin use in the treatment of severe Strongyloides stercoralis infection: Two case reports and a discussion of the literature. J Antimicrob Chemother.

